# Meating Conflict: Toward a Model of Ambivalence-Motivated Reduction of Meat Consumption

**DOI:** 10.3390/foods11070921

**Published:** 2022-03-23

**Authors:** Shiva Pauer, Bastiaan T. Rutjens, Matthew B. Ruby, Grischa Perino, Frenk van Harreveld

**Affiliations:** 1 Department of Social Psychology, University of Amsterdam, 1001 NK Amsterdam, The Netherlands; b.t.rutjens@uva.nl (B.T.R.); f.vanharreveld@uva.nl (F.v.H.); 2 School of Psychology and Public Health, La Trobe University, Melbourne 3086, Australia; m.ruby@latrobe.edu.au; 3 Faculty of Business, Economics and Social Sciences, University of Hamburg, 20146 Hamburg, Germany; grischa.perino@uni-hamburg.de; 4 National Institute for Public Health and the Environment (RIVM), 3721 MA Bilthoven, The Netherlands

**Keywords:** ambivalence, behavioral change, food choice, information-seeking, meat

## Abstract

An increasing number of people are concerned about eating meat, despite enjoying doing so. In the present research, we examined whether the desire to resolve this ambivalence about eating meat leads to a reduction in meat consumption. Our model of ambivalence-motivated meat reduction proposes that the pervasive nature of evaluative conflict motivates meat avoidance, and we highlight two potential mechanisms involved: the anticipation of ambivalence reduction through behavioral change, and information seeking for contents that facilitate meat reduction. Study 1 drew on a cross-sectional 6-day food diary with 7485 observations in a quota sample to investigate why meat-related ambivalence arises and to demonstrate the correlation of ambivalence with meat reduction. Two experiments investigated the causal direction of this association by showing that ambivalence-induced discomfort motivated participants to eat less meat when they introspected on their preexisting incongruent evaluations (Study 2 and 3), which was mediated by the aforementioned mechanisms involved (Study 3; preregistered). The studies utilized diverse samples from Germany, England, and the US (total *N* = 1192) and support the proposed model by indicating that behavioral change is an important coping strategy to resolve ambivalent discomfort in the context of meat consumption. Our model of ambivalence-motivated meat reduction contributes to theorizing on the consequences of ambivalence and the psychology of (not) eating meat.

## 1. Introduction

Meat consumption is a cherished behavior around the world [1]. Many people value the nutritional density, joy, and sociability associated with meat [2,3]. At the same time, an increasing number of them are concerned about the detrimental effects of meat consumption on human health, farmed animals, and the environment [3,4,5]. Indeed, the average meat intake substantially exceeds the recommended maximum intake (with considerable cross-country variance), which has been attributed to about 155,000 human deaths in 2017 due to cardiovascular disease, diabetes, and cancer [6]. Additional meat-related health implications include foodborne infections and pathogens as well as antibiotic resistance [7]. Moreover, cutting down on animal products is a highly effective individual climate action [8,9,10,11], and large numbers of people are concerned about animals in animal agriculture [12,13,14,15]. Surveys in the German general public, for instance, have shown that the majority of people judge the prevailing animal production systems to be morally impermissible [16,17]. These contradictory evaluations related to meat can give rise to ambivalence about eating it [18,19,20].

Berndsen and van der Pligt [18] indicated that ambivalence about meat could be widespread in students and negatively correlated with meat consumption above and beyond the effects of meat-related attitudes, beliefs, and perceived social norms. Although there is ample converging evidence for such a correlation of ambivalence with behavioral change (e.g., [21,22,23]), previous studies have not investigated the causal direction and underlying processes.

In the present article, we investigate a novel explanation of reduced meat consumption by addressing the aforementioned research gap and shedding light on how meat-related evaluative conflict can lead to a reduction in meat intake. We draw on previous research on meat consumption and attitudinal ambivalence to propose and test a model of ambivalence-motivated meat reduction. The goal of this research is to gain insight into the causal direction and the pathways of the association between ambivalence and meat reduction.

## 2. Ambivalence toward Meat Consumption

The awareness of conflict about an object (hereinafter: felt ambivalence) is an aversive state that can arise from the coexistence of strong but incongruent evaluations within the attitude (hereinafter: potential ambivalence) [24,25]. Measures of potential ambivalence and felt ambivalence often correlate only moderately [26], reflecting the notion that ambivalence can either be salient or remain in a dormant (and exclusively structural) state. Someone could hold equally strong positive and negative evaluations of an object—e.g., believing that meat has both health benefits and risks—but this potential ambivalence might elicit feelings of conflict only in situations that make the incongruence salient, such as in deciding how much meat to purchase when grocery shopping. The simultaneous accessibility of incongruent evaluations can make felt ambivalence about eating meat salient [27,28,29], for example, when individuals introspect on their ambivalent attitudes toward meat consumption or in food choice contexts (e.g., [19,30,31]). 

The experiences of felt ambivalence can involve multifaceted attitudinal components. Berndsen and van der Pligt [18] investigated the attitudinal drivers of felt ambivalence about meat consumption in terms of hedonic beliefs, health beliefs, environmental beliefs, morality, social norm, and affective beliefs related to meat consumption. They found that these variables, except for hedonic beliefs and social norm, significantly correlated with felt ambivalence, explaining a total of 30% of the variance in felt ambivalence. More comprehensively, Buttlar et al. [32] investigated the experiences that constitute felt ambivalence about meat consumption and developed a meat ambivalence questionnaire. They found five factors that compose the felt ambivalence due to the animal origins of meat, sustainability considerations, the sociability of meat-related food choices, health considerations, and sensory experiences. In sum, felt ambivalence about meat consumption can occur in a variety of facets that involve especially (inter)personal and moral experiences.

The frequency with which felt ambivalence about meat consumption arises from incongruent evaluations might depend on cultural differences as well as social context (see [33,34]). Previous research has suggested that the presence of people who follow a meat-free diet could make an individual’s attitudinal inconsistency salient [35], such as when an individual strategically expresses ambivalence to enhance their social image [34]. However, there may be considerable variation in the prevalence of such elicitors of meat ambivalence across cultures [3]. For example, meat consumption is a relatively stigmatized behavior in India, with the consequence that social influence during meat consumption could elicit felt ambivalence more frequently than in other countries [36,37]. The magnitude of felt ambivalence about meat could also depend on regional and cultural differences in variables such as the prevalence of meat-related health problems, frequency of exposure to animal slaughter, masculinity, and culinary preferences [38,39,40]. Certain other facets of meat ambivalence seem relatively universal in humans, such as pathogen disgust related to meat, empathy toward farmed animals, or psychological and somatic sensations during the digestion of meat [41,42,43,44]. As such, meat ambivalence could exist in a variety of people across the world, albeit with considerable cultural differences in its elicitors and magnitude. Previous research has indicated the existence of meat-related conflict in a diverse set of countries, including Brazil, China, Ecuador, France, and the USA [3,38,45].

Moreover, the association of potential ambivalence with felt ambivalence may be attenuated by coping strategies that aim to maintain meat consumption despite potential ambivalence [46,47,48]. For example, individuals sometimes strategically dissociate meat from its animal origin, which could decrease the magnitude of felt ambivalence even if they hold incongruent evaluations [49,50]. Congruently, individual differences in meat-related moral disengagement [51,52] may influence the elicitation of felt ambivalence. Previous research has indicated that, for instance, gender and age affect the extent to which people utilize coping strategies like moral disengagement [53,54,55,56]. Taken together, the experience of felt ambivalence about meat consumption encompasses multifaceted inconsistent evaluations with embedment in an individual’s situational and sociodemographic context.

## 3. Ambivalence-Motivated Meat Reduction

Felt ambivalence about meat consumption has been found to have moderate to large correlations with intentions to eat less meat and self-reported meat reduction [18,21,57]. A reason why ambivalence could motivate behavioral change lies in the stability and aversiveness of ambivalent attitudes. Ambivalence toward food and personal goals can be chronic, in many cases lasting longer than a year [22,58], even though the experience of ambivalence is an aversive state that has detrimental effects on well-being [59,60,61]. In line with this assertion, recent research demonstrated that felt ambivalence can come to light when judging pictures of meat [19,62], and such visual stimuli are ubiquitous, for instance in supermarkets. Another elicitor of felt ambivalence is food choice [63], because the experience of ambivalence particularly arises when a decision has to be made [61,64]. Chronic ambivalence about meat therefore involves the aversive recurrence of experiences of ambivalence in daily life situations.

The pervasiveness of ambivalence toward meat indicates that it can be difficult to restore a non-ambivalent attitude toward it. Specifically, alleviating ambivalent discomfort through coping strategies that may frequently be involved in maintaining meat consumption after attitude-behavior conflicts (i.e., meat-related cognitive dissonance [46,65]) would afford only temporary relief to individuals once they have developed a chronically recurring awareness of the conflict. For example, situational underreporting of one’s meat consumption as a response to experiences of meat-related threat [35,66] will not resolve potential ambivalence in the long term or prevent felt ambivalence from chronically recurring. Likewise, cultural adaptations that can prevent the experience of conflict by facilitating the dissociation of meat from its animal origins [46,67] apparently afford insufficient relief to people who are ambivalent, otherwise they should not be chronically ambivalent to begin with. Taken together, the chronic nature of ambivalence indicates that ambivalent people sometimes have difficulties in resolving inconsistencies in a way that restores a non-ambivalent attitude toward meat consumption.

In the absence of effective mechanisms to regain a stable non-ambivalent attitude and avert experiences of ambivalence from recurring, one way to nonetheless reduce the aversive recurrence of felt ambivalence could be avoiding the ambivalent object, i.e., avoidance of meat. According to pervasive models of ambivalence [25,68], avoiding the elicitors of felt ambivalence could be a coping strategy that facilitates the regulation of ambivalence. In line with this assertion, research on choice conflict showed that people will avoid decisions in conflicted choice contexts more often than in unconflicted contexts [69,70,71]. A similar avoidance mechanism might arise from ambivalence. Specifically, the anticipation of ambivalence reduction [72,73] could drive the avoidance of stimuli and situations that elicit felt ambivalence, with the aim to alleviate ambivalence-induced negative affect [68]. The avoidance of meat consumption may thus be perceived as an effective means to reduce the aversive recurrence of felt ambivalence.

**Figure 1:**
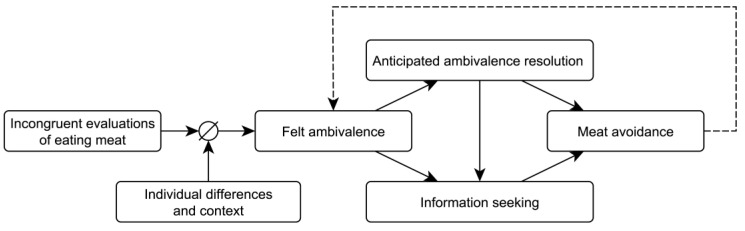
The model of ambivalence-motivated meat reduction.

Above, we argued that the pervasive nature of ambivalence motivates the avoidance of meat consumption through an anticipated reduction of ambivalence. Anticipated ambivalence reduction could also motivate people to seek information about how to eat less meat in order to facilitate successful ambivalence reduction. There is ample evidence that ambivalence can impact on information seeking more generally (e.g., [74,75,76]), which has been conceptualized to be driven by the desire to resolve ambivalence and regain a less ambivalent attitude [68]. Clark et al. [72], for instance, showed that ambivalence led to effortful information processing only if participants believed the offered information could reduce felt ambivalence. In particular, novel information about how to eat less meat is arguably instrumental in coping with barriers to eating less meat [77,78], such as lack of cooking skills [79]. Anticipated ambivalence resolution through meat reduction may therefore motivate seeking out information that facilitates plant-based food choices. In the current research, we conducted three studies with the goal to test our proposed model of ambivalence-motivated meat reduction (see Figure 1). The model addresses the research gap on how ambivalence toward meat consumption leads people to eat less meat. We expected that the awareness of evaluative conflict about eating meat primarily arises from strong but incongruent evaluations of meat. However, context variables (i.e., cultural, situational, and social context) and individual differences may attenuate the association of felt with potential ambivalence. The present research focuses especially on sociodemographic variables, as previous research has often relied entirely on student samples. As a consequence of chronically felt ambivalence, we expect that people avoid meat due to the desire to avert the negative affect of felt ambivalence. Specifically, we propose that the anticipation of ambivalence resolution through meat avoidance motivates behavioral change. This anticipation may increase information seeking to facilitate meat avoidance. Meat ambivalence should also directly motivate information seeking to reduce the ambivalence and gain confidence in evaluating meat consumption.

The hypotheses and analytic plans of the studies reported in this article were specified before the data were collected, if not stated otherwise. We report all data exclusions, experimental manipulations, measures, and sampling procedures.

## 4. Study 1

Study 1 aimed to examine the prevalence of meat ambivalence, its association with meat consumption, and the roles of sociodemographic variables in the proposed model of ambivalence-motivated meat reduction. To this end, a six-day food diary with non-deceptive obfuscation assessed meat consumption in a quota sample that roughly represents the German population of adults. We sought to distinguish the effects of strong but incongruent evaluations of eating meat (i.e., potential ambivalence) and awareness of evaluative conflict (i.e., felt ambivalence) in the association of ambivalence with meat reduction. Our central tenet is that the aversiveness of felt ambivalence [61,80] and its motivational consequences [25,68] are the primary drivers of meat avoidance, given that they motivate people to avert the recurrence of ambivalent discomfort. However, instead of felt ambivalence motivating meat reduction, the barriers to eating less meat (see [78]) could elicit felt ambivalence in people who attempt to eat less meat. To rule out this alternative explanation, we controlled for perceived behavioral control over meat consumption.

## 5. Method

### 5.1. Procedure

An initial sample of 1695 participants was drawn from the online panel of Kantar, a market research company. A screening survey assessed their demographic characteristics and diets (e.g., gluten-free, vegetarian, halal, alcohol-free). We invited only people who indicated that they eat meat and were willing to take all parts of the diary study. The company balanced the demographics according to the German population statistics. On Sunday, 25 November 2018, 882 participants started the first 3 days of the digital diary reports. Subsequently, participants were randomly assigned to read one out of three 100-word paragraphs (as interventions for meat reduction) or a control condition (about unrelated health issues of children). Before that, participants read an article to distract from the topic of meat consumption (i.e., about everyday life contexts of meal intake). These articles were part of an unrelated study that will be published separately—they did not influence the present findings as shown by pre-post and between-group comparisons (see Supporting Information). The remaining 3 days of diary reports were administered starting on the subsequent Sunday. Afterwards, a final questionnaire assessed psychological variables.

### 5.2. Participants

A total of 555 participants completed all parts of the study. This corresponds to 62.9% of the people who started the diary study. Participant characteristics were relatively representative of the German population of adults in terms of age, gender, education, and region; there were no significant differences between the initial and final sample (see Table 1), *ds* < 0.10, *ps* > 0.05. People who followed a vegetarian or vegan diet were not invited to the diary study as they could not further reduce their meat consumption. Participants who completed the study received €22.

### 5.3. Materials

***Meat Consumption Frequency.*** A 6-day food record [81] assessed meat consumption and other food choices, resulting in a total of 7485 meal observations. We incorporated non-deceptive obfuscation to distract from the topic of meat consumption to reduce non-target effects such as demand effects (i.e., changes in the behavior due to cues in the study design) [82] and selection bias (i.e., the study description influence whether people participate or not) [83]—these non-target effects can inflate associations with psychosocial determinants [84]. Specifically, participants had to select boxes of the ingredients included in their meals on a list of 13 categories—e.g., meat, potatoes, fruit. A total of eight additional questions were assessed as distractors from food choice or for an unrelated study, such as activities and intake of fluids (see Supporting Information for the questionnaire). Participants were asked to report a minimum of two meals per day immediately after the meal. Meat consumption frequency was calculated as the proportion of meals that included meat relative to the total number of meals reported by an individual.

***Felt Ambivalence.*** Awareness of evaluative conflict toward eating meat was measured with one item adapted from the Felt Ambivalence Questionnaire [24,85]: “To what degree do you experience conflicting thoughts and/or feelings regarding eating meat?” The 5-point response scale included two labels (1—*not at all* and 5—*very much*).

***Potential Ambivalence, negativity, and positivity.*** Two split semantic differential scales [86] measured positive and negative associations to eating meat: “How positive [/negative] are your thoughts and/or feelings regarding meat consumption?” (*r* = −0.71). The 5-point response scales ranged from 1 (*not at all positive*[/*negative*]) to 5 (*extremely positive*[/*negative*]). We calculated Thompson et al. [87]’s score for potential ambivalence from the intensity of the positive (P) and negative (N) associations and subtracted their congruences: (P + N)/2 − |P − N|. The resulting scores range from −1 to 5, high scores indicate both strong and opposed associations. For example, a score of −1 reflects the largest distance between positive and negative associations.

***Perceived Behavioral Control*.** One item measured the perceived behavioral control [88] that someone may experience in eating meat: “I can control whether and how much meat I eat.” The 5-point response scale ranged from 1 (*completely agree*) to 5 (*completely disagree*).

***Sociodemographics*.** Sociodemographic information was assessed in 15 questions (see Supporting Information), for example, number and age of children, employment and health status, and social context (i.e., the number of vegetarian and vegan friends and family members).

## 6. Results and Discussion

All regression coefficients are in standardized format. CIs are based on 5000 bootstrap samples in mediation analyses, with the mediation/indirect effects reflecting the multiplied standardized coefficients of the associations between variables (see [89]).

### 6.1. Prevalence and Determinants of Meat Ambivalence

A total of 66.8% of participants indicated that they experienced at least some meat-related conflict, as they selected a response option above the scale minimum of 1 that was labelled as “not at all” conflicted. A one-sample *t*-test revealed that the average felt ambivalence was significantly below the scale midpoint of 3 with considerable variance between individuals, *M* = 2.39, *SD* = 1.18, *t*(554) = t, *t*(554) = 12.24, *p* < 0.001, *d* = 0.52. Exploratory analyses showed that felt ambivalence significantly correlated with five sociodemographic variables out of 15 (see Table 2). After stepwise exclusions in multiple regression, only gender (1—female, 0—male), β = 0.17, *p* < 0.001, and social context, β = 0.24, *p* < 0.001, remained significant predictors, *R*^2^ = 0.08. Controlling for meat consumption frequency, the effects of gender, β = 0.15, *p* < 0.001, and social context, β = 0.21, *p* < 0.001, remained significant.

To our knowledge, this is the first data to investigate the prevalence of felt ambivalence in the general public. While the prevalence of felt ambivalence may be lower than that in student samples (e.g., [18]), the present findings support the assumption that it is a relatively widespread phenomenon [46,90] in our sample but with considerable variance. That is, most participants reported at least some felt ambivalence, whereas a substantial part of the sample reported a complete absence of felt ambivalence. As a consequence, the total mean of felt ambivalence was below the scale midpoint with a small to moderate difference. Taken together, the findings underline the relevance of the question of why some people (do not) feel conflicted about meat consumption. Individuals who are female or have more vegans and vegetarians in their social circle reported greater felt ambivalence. Interestingly, the gender difference holds above the effect of gender on meat consumption indicated in previous research [56,91,92,93], suggesting that gender could influence felt ambivalence due to gender differences in coping strategies like moral disengagement. The effect of social context is in line with previous research on interpersonal predictors of ambivalence [33,34] and highlights that sociability could be a crucial elicitor of meat ambivalence.

**Figure 2:**
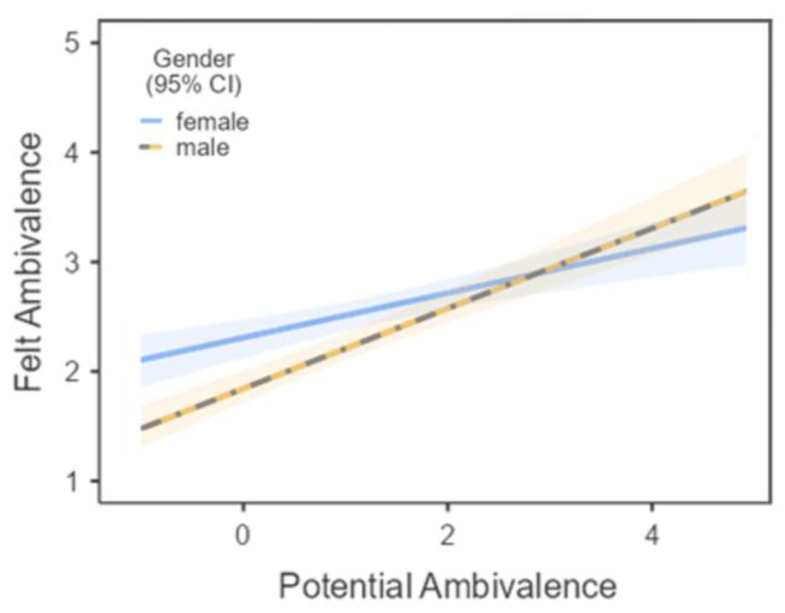
Gender and potential ambivalence interact to predict felt ambivalence about meat consumption.

**Figure 3:**
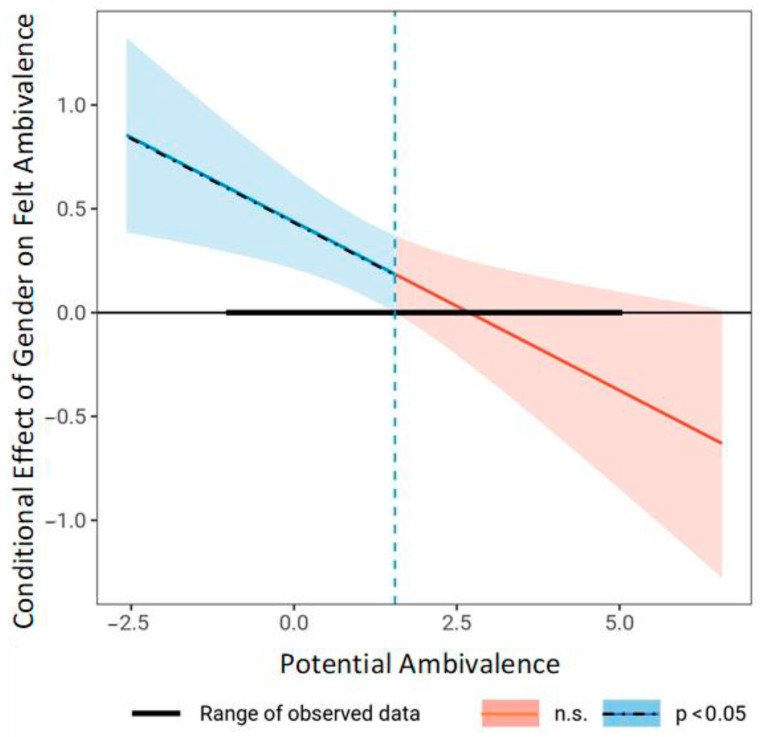
Johnson–Neyman plot of the association of gender with felt ambivalence at values of potential ambivalence about meat consumption. The dotted vertical line indicates the border of the significance region at a value of potential ambivalence of 1.55, with 63.65% of participants scoring below that value.

Next, multiple regression analyses investigated moderators of the association of potential ambivalence (i.e., the coexistence of strong but incongruent evaluations about meat consumption) with felt ambivalence in Process [89]. As predicted and in line with previous research (see [25]), the association of potential ambivalence with felt ambivalence was only moderate (see Table 2). We therefore investigated the roles of gender and social context in moderation analyses that indicated interaction effects of the two variables with potential ambivalence on felt ambivalence. The association of potential ambivalence with felt ambivalence was more pronounced in males, β = 0.49, than females, β = 0.27, *ps* < 0.001, with a significant interaction of gender and potential ambivalence, β = −0.22, *p* = 0.006 (see Figure 2). There were significant gender differences in felt ambivalence at low levels of potential ambivalence, β = 0.51, *p* < 0.001, and mean potential ambivalence, β = 0.23, *p* = 0.003, but not at high potential ambivalence, β = −0.04, *p* = 0.72 (see Figure 3). Likewise, social context and potential ambivalence interacted, β = −0.17, *p* = 0.013, such that the association of potential ambivalence with felt ambivalence was more pronounced at low SC, β = 0.41, and mean social context, β = 0.36, than at high social context, β = 0.25, *ps* < 0.001. Social context significantly predicted felt ambivalence only at low levels of potential ambivalence, β = 0.53, *p* < 0.001, and mean potential ambivalence, β = 0.32, *p* < 0.001, but not at high potential ambivalence, β = 0.10, *p* = 0.18. There were no significant interaction effects on felt ambivalence by social context and gender or by social context, gender, and potential ambivalence, βs < 0.05, *ps* > 0.58.

The findings substantiate our model of ambivalence-motivated meat reduction (see Figure 1) by highlighting that there are individual differences in the extent to which potential ambivalence predicts felt ambivalence. The gap in the merely moderate association of potential ambivalence with felt ambivalence seems to depend in part on female gender and social context increasing the likelihood of experiencing felt ambivalence in individuals who show relatively low levels of potential ambivalence. Interestingly, gender and social context did not significantly impact felt ambivalence at high potential ambivalence, which will be elaborated upon in the General Discussion section.

### 6.2. Ambivalence and Meat Consumption

On average, 46.3% of the reported meals included meat, *SD* = 24.18, *Mdn* = 45.4. This estimate does not necessarily reflect the exact true frequency of meat consumption, as some participants could have underreported certain meal types. On average, they reported 2.25 meals per day, 35.4% of which involved breakfast, 33.0% lunch, and 31.6% dinner. The proportion of meat consumption ranged from 31.7% at breakfast to 59.9% at dinner. As such, underreporting of lunch or dinner might slightly increase error variance in predicting meat ambivalence, which would result in a modest underestimation of the true coefficients.

**Figure 4:**
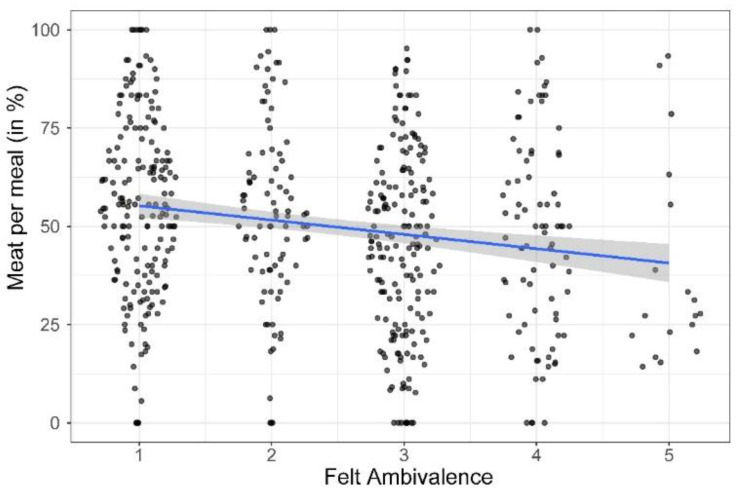
Scatterplot of the association of felt ambivalence with meat consumption.

Felt ambivalence correlated negatively with meat consumption frequency, *r*(553) = −0.18, *p* < 0.001 (see Figure 4). Unlike previous research [21], there were no significant curvilinear associations as indicated by planned contrasts, *ps* > 0.05. Controlling for covariates that remained significant after stepwise exclusions (i.e., gender, age, household size, and education, *ps* < 0.05) the partial correlation of felt ambivalence and meat consumption remained significant, *r*(547) = −0.14, *p* = 0.001. An exploratory multiple regression tested whether the effect of felt ambivalence on meat consumption remained significant after controlling for the evaluative components of the attitude that form the ambivalence, given that they can influence both felt ambivalence and meat consumption. The effect of felt ambivalence turned nonsignificant after including negativity, positivity, and potential ambivalence, β = −0.02, *p* = 0.674. Note that this test has been argued to be invalid as the covariates partial out parts of the target effect [94]. Specifically, the causal effects of felt ambivalence on affective states [61,95] play a crucial role in our conceptual model by affecting the desire to reduce ambivalence-induced discomfort through behavioral change. The lack of conclusiveness of this test highlights a need to gain better insight into the isolated role of felt ambivalence using experimental methods (see Study 2 and 3).

Finally, we tested the role of felt ambivalence in meat reduction in relation to potential ambivalence and perceived behavioral control. As predicted, a mediation analysis in Process (model 4) [89] showed that there was an indirect effect of potential ambivalence on meat consumption through felt ambivalence, β = −0.05, 95% CI [−0.09, −0.01], with a significant direct effect of felt ambivalence on meat consumption β = −0.12, *p* = 0.008. Next, multiple regression analyses supported the prediction that the aversive nature of ambivalence can drive meat reduction above the potential effect of a lack of perceived behavioral control in meat reduction on felt ambivalence, as the association of felt ambivalence with meat consumption remained significant above and beyond the effect of perceived control on meat consumption (see Table 3).

These findings replicate a correlation of felt ambivalence with meat consumption [18] using a high-quality sample and measure of meat consumption frequency that reduced self-selection and recall biases, for instance. Moreover, in line with our model of ambivalence-motivated meat reduction, the effect of potential ambivalence on meat reduction was mediated by felt ambivalence. In addition, felt ambivalence seems to lead to meat reduction rather than vice versa through the difficulty of reducing meat consumption (i.e., perceived behavioral control). These findings support our assumption that the aversive nature of experiences of ambivalence may motivate meat reduction.

## 7. Study 2

Study 1 suggests that the negative association of meat-related ambivalence with meat consumption might be driven by the aversive nature of ambivalence. Study 2 sheds light on the process through which ambivalence and meat avoidance are associated by experimentally manipulating felt ambivalence. Based on our model of ambivalence-motivated meat reduction (see Figure 1), we hypothesized that situations that bring to mind the pervasive nature of awareness of ambivalence (i.e., felt ambivalence) should increase meat avoidance. We adopted a common procedure to make felt ambivalence salient by increasing the simultaneous accessibility of incongruent evaluations [27,28]. Specifically, we asked participants to introspect on the inconsistent evaluations (i.e., potential ambivalence) that they hold (see [29]). This manipulation elicits felt ambivalence without necessarily impacting on the magnitude of potential ambivalence, as inconsistent evaluative components are relatively stable, whereas the magnitude of felt ambivalence depends on situational contexts [25,27]. As mentioned earlier, however, the elicitation of felt ambivalence may cause aversive states, such as anticipated regret, that drive the desire to resolve ambivalence (see [25]), such as through ambivalence-induced negative judgments of the ambivalent object [95]. Therefore, we included manipulation checks to guarantee that ambivalence salience increased felt ambivalence.

We measured behavioral intentions as the proximal determinant of meat avoidance. We deemed intentions to be a sufficiently adequate proxy based on meta-analytic evidence on experimentally-induced intentions often leading to actual behavioral change [96]. We included a reverse-coded intention item to account for the possibility that meat-related conflict could be resolved through whatever coping opportunity is afforded, such as overreporting of intentions (see [45,97]).

In addition to a self-report measure of intended meat avoidance as the main outcome, we administered a charitable donation task about a central driver of ambivalence toward meat consumption, namely the animal origins of meat [18]. This task was primarily included as it corresponds to a personally relevant choice instrument that reduces hypothetical bias, which can lead to overreporting of intentions, for instance [98,99]. As such, an experimental effect on this task would provide converging evidence for ambivalence-motivated meat reduction. Moreover, a significant experimental effect on both intention and donation would rebut the possibility that participants cope with the aversive feeling of conflict merely through resorting to the affordance that is provided first [97,100]. Taken together, we assumed that people effortfully attempt to avert ambivalence from recurring, which includes increased engagement with both meat reduction and donation for societal change to resolve the elicitors of ambivalence toward meat consumption.

## 8. Method

### 8.1. Participants and Design

The final sample of 189 participants mainly from North America (90.0%, *M*_age_ = 35.81, *SD* = 11.28, 63.5% female, 54.5% with university degrees) was recruited through the online-recruitment panel MTurk. We excluded 13 participants (6.4%) who failed predetermined quality checks by duplicate participation or failing a simple attention check (i.e., “please select ‘very much’”) [101]. As predetermined, a total of 12 people who followed meat-free diets were excluded from data analyses given that they could not further reduce their meat consumption. The study employed a two-group between-subjects design. We targeted at an a priori determined sample size of 172 valid cases to detect an expected effect size of *d* = 0.50 [27] at an alpha level of 0.05 with a power of 90%.

### 8.2. Materials and Procedure

Participants were invited to a study on “everyday life behavior”. They were randomly assigned to an introspective ambivalence manipulation task about eating meat or an unrelated control issue (vaccination). Subsequently, they answered questions on potential ambivalence, felt ambivalence, intentions, charitable donation, and sociodemographics.

***Ambivalence Manipulation:*** We elicited ambivalence about eating meat using an introspective ambivalence manipulation procedure (adapted from [29,102]). This ecologically valid procedure has frequently been used to make felt ambivalence salient by having participants introspect on their preexisting incongruent evaluations or personal experiences, and thus increase the simultaneous accessibility of attitudinal inconsistency (see [27] for a meta-analysis, [28]). Participants were asked to describe two personally held reactions in favor of and two reactions opposed to meat consumption or vaccination in the control group:

“We would like to know your arguments/feelings in favor of and against meat consumption [vaccination]. Please think about meat consumption [vaccination] and, firstly, write down your 2 strongest arguments/feelings in favor of meat consumption [vaccination] and, secondly, your 2 strongest arguments/feelings against meat consumption [vaccination]. The aim of this task is to assess your personal experiences and preferences.”

***Felt Ambivalence:*** Awareness of ambivalence about eating meat was measured using the Felt Ambivalence Questionnaire [24], which is based on the tripartite model of attitudes (e.g., [103]). The instrument measures the affective, behavioral, and cognitive components of experiences of ambivalence. It encompasses three items that read “Toward eating meat I…” with 10-point response scales ranging from 1 (*feel no conflict at all*/*feel no indecision at all*/*have completely one-sided reactions*) to 10 (*maximum conflict*/*maximum indecision*/*completely mixed reactions*). Internal reliability was good, α = 0.87.

***Potential Ambivalence, Negativity, and Positivity:*** These variables were measured as in Study 1 but on 10-point response scales (*r* = −0.66).

***Behavioral Intention:*** Intention to reduce meat consumption was assessed on two items (adapted from [104,105]): “I intend to reduce my meat consumption next week,” and “I will maintain my current levels of meat consumption in the future” (reverse-coded) on scales from 1 (*strongly disagree*) to 10 (*strongly agree*), *r* = 0.76.

***Charitable Donation:*** Participants were told that they qualified for a bonus payment drawing of $10 for passing the attention check item presented on the preceding page. They could type in any donation amount that we would donate if they did not want to receive the complete bonus. The charity was “an animal advocacy organization trying to reform the way farmed animals are treated” (see Supporting Information for details).

***Meat Consumption Frequency:*** For the purpose of excluding strict meat avoiders and to rule out heterogeneous treatment effects, participants were asked to write down either the average times per day or the days per week, month, or year that they consumed meat (see Supporting Information).

## 9. Results and Discussion

Regression coefficients are in standardized format. CIs are based on 5000 bootstrap samples in mediation analyses. There were no significant confounders or heterogeneous treatment effects of the experimental manipulation such as due to gender, age, or education, *ps* > 0.17.

Firstly, manipulation checks showed that meat-related ambivalence salience successfully elicited felt ambivalence toward eating meat without relevant effects on the evaluative components that form the ambivalence. Specifically, a between-subjects ANOVA indicated that felt ambivalence was higher in the ambivalence salience condition (*M* = 4.70, *SD* = 2.47, *n* = 100) compared to the control (*M* = 3.87, *SD* = 2.45, *n =* 89), *F*(1, 187) = 5.28, *p* = 0.023, *d* = 0.34. Controlling for potential ambivalence as well as negative and positive associations to meat, the effect of ambivalence salience on felt ambivalence remained significant, *F*(1, 184) = 4.51, *p* = 0.035, *d* = 0.31. As predicted, there was no significant effect of ambivalence salience on potential ambivalence, *F*(1, 187) = 0.24, *p* = 0.625, *d* = 0.07, or positive evaluation, *F*(1, 187) = 0.14, *p =* 0.709, *d* = 0.06. The experimental effect on negative evaluation of meat reached significance, *F*(1, 187) = 4.87, *p =* 0.029, *d* = 0.32, but, crucially, turned nonsignificant, *F*(1, 186) = 1.12, *p =* 0.291, *d* = 0.16, after including felt ambivalence as a covariate. The manipulation checks therefore indicate that ambivalence salience successfully elicited felt ambivalence by making participants’ preexisting evaluative incongruence salient without increasing potential ambivalence, in accordance with previous research [27,28,29]. Importantly, the effect of the manipulation on felt ambivalence remained significant after controlling for potentially confounding effects of potential ambivalence, negativity, and positivity. Congruently, the effect of the manipulation on negativity was completely explained by felt ambivalence—in other words, the experimentally-elicited felt ambivalence increased ambivalent discomfort, which replicates previous research on ambivalence causing negativity, including anticipated regret and negative judgements [25,61,95]. Thus, the experimental manipulation was successful in eliciting felt ambivalence rather than negativity.

Next, we tested for an experimental effect on the main outcome variable. Ambivalence salience significantly increased behavioral intention to reduce meat consumption (*M* = 4.19, *SD* = 2.72) compared to the control condition (*M* = 3.26, *SD* = 2.27), *F*(1, 187) = 6.42, *p* = 0.012, *d* = 0.37. An exploratory ANCOVA controlled for potentially confounding effects of ambivalence salience on intention through evaluative components that might influence felt ambivalence. That is, the effect of ambivalence salience on intention remained significant, F(1, 184) = 4.14, *p* = 0.043, after including potential ambivalence, negativity, and positivity as covariates. Note that this usage of ANCOVAs has been argued to be invalid, given that the covariates partial out parts of the target effect [94], i.e., the causal effects of felt ambivalence on affective states (see [25]). While we deem the manipulation checks more conclusive, the isolated effect of ambivalence salience on intention after controlling for covariates further strengthens confidence in the pattern of findings.

Two ANOVAs tested whether the experimental effect on intention held irrespective of the directions of the two intention items. As expected, there were stronger intentions to reduce meat consumption in the ambivalence salience condition (*M* = 4.15, *SD* = 2.83) compared to the control condition (*M* = 3.11, *SD* = 2.41), *F*(1, 187) = 7.27, *p* = 0.008, *d* = 0.39, and lower intentions to maintain the current level of meat consumption (*M* = 7.77, *SD* = 2.93) compared to the control condition (*M* = 8.60, *SD* = 2.55), *F*(1, 187) = 4.23, *p* = 0.041, *d* = 0.30.

For converging evidence for the experimental effect on motivating behavioral change, we tested whether ambivalence salience increased charitable donation. Surprisingly, donation was not significantly increased by ambivalence salience (*M* = 1.93, *SD* = 2.23) compared to the control condition (*M* = 1.79, *SD* = 2.16), *F*(1, 187) = 0.20, *p* = 0.655, *d* = 0.07.

**Figure 5:**
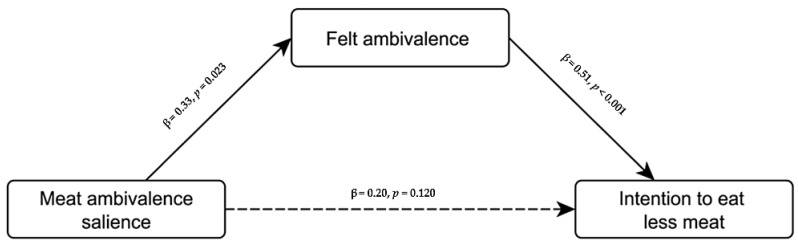
Mediation model of the experimental effect of meat ambivalence salience on intention to eat less meat through felt ambivalence in Study 2. Effect sizes are displayed as standardized direct effects (i.e., isolated from the other effects).

Finally, we tested whether felt ambivalence explained the experimental effect on intention. As hypothesized, a mediation analysis (Process model 4) [89] indicated a positive indirect effect of ambivalence salience on intention through felt ambivalence, β = 0.17, *SE* = 0.08, 95% CI [0.02, 0.32], such that the direct effect of ambivalence salience turned nonsignificant (see Figure 5).

The findings indicate that ambivalence salience increases meat avoidance as an effect of felt ambivalence. This supports the assumption that the aversive nature of meat-related ambivalence can motivate meat avoidance, arguably due to anticipated ambivalence reduction. Contradictory to our predictions, however, there was no significant effect of ambivalence salience on charitable donation, which might possibly be due to offering donation to an animal advocacy NGO instead of a donation opportunity that addresses all prevalent components of meat ambivalence. This limitation will be addressed in Study 3.

## 10. Study 3

In Study 3 (preregistered), we primarily aimed to replicate the experimental effects observed in Study 2 and expand on the mechanisms involved in ambivalence-motivated meat reduction. We preregistered the hypotheses that ambivalence salience increases felt ambivalence toward meat, which predicts meat avoidance through increasing the perceived potential of meat avoidance to resolve the ambivalence. This anticipation of ambivalence resolution, in turn, motivates effortful information-seeking on how to eat less meat (see Figure 1 for a conceptual model). Our central tenet is that the salience of the pervasive experience of ambivalence leads to meat avoidance. This is based on the assumption that alleviating the ambivalent discomfort through other coping strategies, such as moral disengagement (e.g., [106]), would afford only temporary, and thus less effective, coping opportunities once someone has developed a chronically recurring awareness of the conflict. Meat avoidance could be motivated to alleviate the aversive nature of meat ambivalence and prevent feelings of conflict from recurring.

We aimed to directly examine the motivational underpinning of ambivalence-induced meat reduction by assessing the perceived potential of meat avoidance to reduce felt ambivalence. We predicted an indirect effect of ambivalence salience on meat avoidance through this variable. Moreover, this effect could indicate a twofold process toward meat avoidance. Firstly, anticipated ambivalence resolution might directly motivate meat avoidance as a way of reducing chronic ambivalence. It may also motivate effortful information seeking related to meat to regain a non-ambivalent attitude and increase the feasibility of meat avoidance, for instance through learning about plant-based meat-alternatives. As an additional mechanism, Study 3 therefore tested whether anticipated ambivalence resolution predicts meat avoidance through information seeking about meat reduction.

Given that Study 2 did not yield a significant experimental effect on charitable donation, we adjusted the donation instrument to incorporate an NGO that addresses the most prevalent components of meat ambivalence. These components were identified in an ancillary content-analysis of the introspective evaluations obtained from Study 2—i.e., health benefits and risks, animal ethics, sustainability, and sensory pleasure (see Supporting Information). As in Study 2, this measure was aimed to provide converging evidence for ambivalence-motivated behavioral change using a performance-based instrument that reduces hypothetical bias [98,99], and rule out the possibility that participants cope with feeling conflicted merely through the affordance that is provided first [97,100].

## 11. Method

### 11.1. Participants

A final sample size of 448 participants from England (*M*_age_ = 35.05, *SD* = 12.16, 58.7% female, 60.7% with university degrees) was recruited through the online recruitment platform Prolific. A total of 20 participants were excluded due to failing preregistered quality checks, namely less than 2 s median speed per item [107] and attention checks (i.e., noncompliance in an open-ended question, and “please select ‘very much’ as a check of your attention?”). As preregistered, a total of 12 people who followed meat-free diets were not included in the analyses as they could not further reduce their meat consumption.

We preregistered a target sample size of 452 valid cases based on a two-group between-subjects design. This sample size provides 80% power to detect effect sizes of *ds* > 0.265 on the behavioral outcomes at an alpha level of 0.05. The preregistration document can be found at osf.io/7w69j (last accessed on 13 January 2022).

### 11.2. Materials and Procedure

Participants were randomly assigned to an introspective ambivalence manipulation task concerning meat consumption or an unrelated control topic (going to the cinema). Due to a technical problem, 222 participants had to be excluded from the analyses and were replaced by collecting data from the same number of new participants. Specifically, they were not presented with one of the preregistered experimental manipulations due to a coding error. Consequently, the randomization allocation to the two experimental groups had to be adjusted during data collection, which is a permissible change [108] as the complete dataset was recorded within 3 h without inducing relevant allocation biases in terms of sociodemographic differences (see Supporting Information for details).

***Ambivalence Manipulation & Felt Ambivalence:*** We elicited felt ambivalence about eating meat as in Study 2 (with streamlined and revised instructions, see Supporting Information). Felt ambivalence was measured as in Study 2 but on 7-point response scales (α = 0.84).

***Potential Ambivalence, Negativity, Positivity:*** As in Study 1 and 2, two split semantic differential scales [86] measured positive and negative associations to eating meat on 7-point response scales ranging from 1 (*not at all positive*[/*negative*]) to 7 (*extremely positive*[/*negative*]): “Considering only the positive[/negative] aspects of meat consumption, while ignoring the negative[/positive] aspects, how positive[/negative] are your thoughts and/or feelings regarding meat consumption?” (*r* = −0.28). Thompson et al. [87]’s score of potential ambivalence was calculated as in Study 1.

***Anticipated Ambivalence Resolution:*** We measured the perceived potential of meat avoidance to reduce felt ambivalence with three questions (adapted from [72]) that are based on a tripartite model of attitudes (e.g., [103]): “To what extent do you believe that choosing to eat less meat would help you resolve any [conflicted feelings, indecision, mixed reaction] you may have about eating meat?” The response scales ranged from 1 (*not at all*) to 7 (*very much*), α = 0.95.

***Behavioral Intention:*** Intention to eat less meat was assessed on two items (adapted from [104,105]): “To what extent are you willing to reduce your meat consumption?” and “Do you intend to follow a more plant-based diet within the next year?” (α = 0.88).

***Charitable Donation:*** Donation was assessed as in Study 2, but the charity was “The Good Food Institute, an NGO that develops healthy and sustainable alternatives to animal-based meat that make plant-based food choices easier”.

***Information Seeking:*** Information seeking about plant-based foods was measured in a choice setting. Participants were told that they would read one out of three articles on the next page, which would be chosen based on their interest in the three topics: “The easiest ways to transition to reduced-meat diets”, “How to prepare tasty plant-based meals”, “What to eat more of if you’re eating less meat”. The response scales ranged from 1 (*not at all*) to 7 (*very much*), α = 0.84. All participants were subsequently presented with recommendations for plant-based eating from Health Canada and reading time was assessed by TaskMaster [109]. However, there were no significant effects on reading time due to low compliance (see Supporting Information).

***Meat Consumption Frequency:*** We adjusted the assessment of meat consumption frequency in order to reduce biased recall of the behavior (see [110]). Participants entered the number of “lunch and dinner meals that you usually eat in a regular week, for instance last week, that contain meat” (adapted from [111]) (see Supporting Information for details).

## 12. Results and Discussion

In this section, we firstly address confounders and heterogeneous treatment effects, followed by a report of the effects of the experimental manipulation of felt ambivalence and, thirdly, mediation analyses.

### 12.1. Confounders and Heterogeneous Treatment Effects

Gender and education emerged as significant confounders (*ps* < 0.05) in most treatment effects as they were associated with meat ambivalence and meat consumption, in line with Study 1 and meta-analytic evidence [27]. Exploratory moderation analyses revealed that there were no significant heterogeneous treatment effects, *ps* > 0.08. We therefore report all findings controlling for these covariates if not stated otherwise, in accordance with the preregistration.

The absence of significant confounding effects in Study 2 might possibly be explained by a larger and more educated, but less female sample in Study 3 (see [27]). The absence of significant heterogeneous treatment effects replicates Study 2 but not previous research (e.g., [112]). One potential explanation might be a low experimenter demand effect in our experimental procedure, whereas merely announcing a video presentation related to farmed animal suffering has been shown to diminish meat commitment in females [66].

### 12.2. Experimental Effects

As in Study 2, manipulation checks showed that meat-related ambivalence salience successfully elicited felt ambivalence without relevant experimental effects on the evaluative components that form the ambivalence. An ANCOVA showed that felt ambivalence was higher in the meat ambivalence salience condition (*n* = 232) compared to the control condition (*n* = 216), *M*_adj_(ambivalence salience) = 3.62, *M*_adj_(control) = 3.14, *SE* = 0.09, *F*(1, 444) = 15.19, *p <* 0.001, *d* = 0.35. Additional tests (not preregistered) revealed that the effect of ambivalence salience on felt ambivalence remained significant after controlling for potential ambivalence as well as its components, i.e., negative and positive associations to meat consumption, *F*(1, 443) = 11.79, *p* < 0.001. There was no significant effect of ambivalence salience on potential ambivalence, *F*(1, 444) = 2.11, *p =* 0.147, *d* = 0.14, or positivity, *F*(1, 444) = 0.16, *p =* 0.694, *d* = 0.04. The experimental effect on negativity reached significance, *F*(1, 444) = 4.04, *p =* 0.045, *d* = 0.19, but turned nonsignificant, *F*(1, 444) = 0.01, *p =* 0.919, *d* = 0.01, after including felt ambivalence as a covariate.

These manipulation checks replicate results from Study 2 by indicating that ambivalence salience successfully elicited felt ambivalence by making participants’ preexisting evaluative incongruence salient without increasing potential ambivalence, in line with previous research [27,28,29]. Again, the effect of the manipulation on felt ambivalence remained significant after controlling for potentially confounding effects of potential ambivalence, negativity, and positivity. Congruently, the effect of the manipulation on negativity was completely explained by felt ambivalence—in other words, the experimentally-elicited felt ambivalence increased ambivalent discomfort, which also replicates previous research on ambivalence causing negative affect and judgment [25,61,95]. Thus, the experimental manipulation was successful in eliciting felt ambivalence rather than negativity.

Next, we tested the experimental effects on the main outcome variables. As hypothesized, ambivalence salience increased anticipated ambivalence reduction, *M*_adj_(ambivalence salience) = 3.95, *M*_adj_(control) = 3.59, *SE* = 0.12, *F*(1, 444) = 4.66, *p* = 0.031, *d* = 0.20, and information seeking about meat reduction, *M*_adj_(ambivalence salience) = 4.70, *M*_adj_(control) = 4.33, *SE* = 0.11, *F*(1, 444) = 5.81, *p* = 0.016, *d* = 0.22. The experimental effect on intention to eat less meat was marginally significant but in the same direction as the experimental effect on intention in Study 2, *M*_adj_(ambivalence salience) = 4.18, *M*_adj_(control) = 3.90, *SE* = 0.11, *F*(1, 444) = 3.27, *p* = 0.071, *d* = 0.16. Charitable donation was increased without significant confounders, *M*(ambivalence salience) = 2.86, *SD* = 2.72, *M*(control) = 2.29, *SD* = 2.55, *F*(1, 447) = 5.15, *p* = 0.024, *d* = 0.22.

We further examined the role of felt ambivalence in the experimental effects in exploratory and non-preregistered ANCOVAs that aimed to partial out potentially confounding effects on the evaluative components that drive the ambivalence. Specifically, after including potential ambivalence, negativity, and positivity as covariates, the effects of ambivalence salience remained significant for donation, F(1, 443) = 4.46, p = 0.035, and information seeking, F(1, 441) = 4.48, *p* = 0.035. Unlike Study 1, the isolated effect of ambivalence salience did not reach significance for intention, F(1, 441) = 1.16, *p* = 0.283, and for anticipated ambivalence reduction, F(1, 441) = 1.99, *p* = 0.159. Note, however, that this usage of ANCOVAs has been argued to be invalid given that the covariates partial out parts of the target effect [94]. This is because felt ambivalence is an aversive state that influences affective and evaluative processes [61,95], which play a crucial role in our conceptual model by affecting the desire to reduce ambivalence-induced discomfort through behavioral change. In light of the aforementioned and more conclusive manipulation checks, the two significant effects of ambivalence salience after controlling for evaluative components further strengthen confidence in the pattern of findings.

### 12.3. Serial Indirect Effects

**Figure 6:**
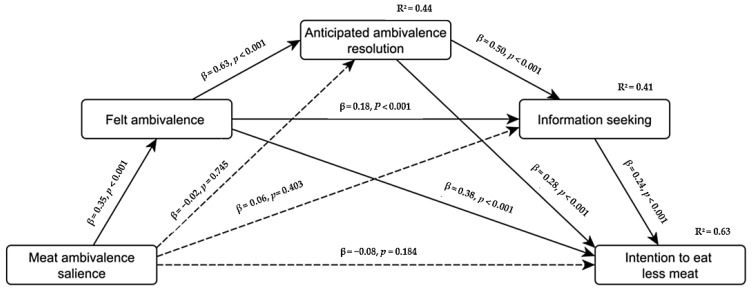
Serial mediation model of ambivalence-motivated intention to eat less meat in Study 3 with an indirect effect of ambivalence salience on intention through felt ambivalence, anticipated ambivalence resolution, and information seeking. Regression coefficients are displayed as standardized direct effects (i.e., isolated from the other effects). *R*^2^ can be interpreted as the percentage of variance explained.

Finally, we tested the pathways through which ambivalence salience is associated with the intention to eat less meat. The following regression coefficients are in standardized format, and mediation analyses are based on 10,000 bootstrap samples run in Process [89]. The preregistered serial mediation model was supported with a positive serial indirect effect of ambivalence salience on behavioral intentions through felt ambivalence, anticipated ambivalence resolution, and information seeking, β = 0.03, *SE* = 0.01, 95% CI [0.01, 0.05] (see Figure 6). The experimental effects of ambivalence salience were successfully explained by the mediators such that they turned nonsignificant. The analysis revealed that the indirect effect solely through felt ambivalence, β = 0.14, *SE* = 0.04, 95% CI [0.06, 0.21], remained significant even after controlling for the other variables in the model. To also probe the other two simple indirect effects of ambivalence salience on intention through each of the two mediators, we firstly run a parallel mediation analysis that revealed significant isolated simple indirect effects solely through anticipated ambivalence reduction, β = 0.05, *SE* = 0.03, 95% CI [0.01, 0.11], or information seeking, β = 0.05, *SE* = 0.02, 95% CI [0.01, 0.10]. As a second and non-preregistered robustness check, two separate simple mediation models showed, again, significant indirect effects of ambivalence salience on intention solely through anticipated ambivalence reduction, β = 0.13, *SE* = 0.06, 95% CI [0.01, 0.25], or information seeking, β = 0.13, *SE* = 0.05, 95% CI [0.02, 0.24]. For converging evidence, we entered charitable donation as a performance-based outcome into the serial mediation model instead of intention, which yielded the same pattern of findings with an indirect effect of ambivalence salience on donation through the three mediators in serial, β = 0.02, *SE* = 0.01, 95% CI [0.01, 0.04].

These findings substantiate our model of ambivalence-motivated meat reduction. The experimentally elicited salience of the aversive nature of meat ambivalence increased both meat reduction intention and charitable donation. This finding reduces the likelihood of non-target effects such as an order effect or hypothetical bias. Moreover, mediation analyses provided initial support for potential roles of anticipated ambivalence reduction and information seeking in how meat ambivalence leads to meat avoidance. While the mediation analyses support our hypotheses, we do not claim that they warrant causal roles of the mediators (see [113]). Likewise, the findings do not rule out alternative serial mediation orders, even though the order highlighted in the present work is based on ample prior research (e.g., [68,72,75,114]).

## 13. General Discussion

The current research investigated how meat-related ambivalence can motivate meat avoidance, and in doing so it makes several important contributions to research on ambivalence and the psychology of (not) eating meat. The studies support the proposed model of ambivalence-motivated meat reduction (see Figure 1), which adds to previous research that has indicated a correlation of ambivalence with self-reported (intention for) meat reduction [18,21,57]. We replicated these correlational studies using a robust measure of meat consumption (Study 1) and provide evidence for the causal direction and potential mechanisms of the association (Study 2 and 3). Taken together, these insights advance the understanding of why people decide to eat less meat—a behavioral change that can substantially increase one’s life expectancy and is relevant to several UN Sustainable Development Goals as well as farmed animal suffering (e.g., [6,7,8,10,115,116,117]). Moreover, our research has implications for designing novel interventions for meat reduction, as discussed below.

The findings of Study 1 revealed that gender and social context moderate the extent to which attitudinal inconsistency is reflected in an awareness of conflict. Specifically, a social circle with a high number of meat avoiders could possibly induce feelings of conflict in meat eaters despite low attitudinal inconsistency to enhance their social image in the social circle [34], and close others who follow a meat-free diet could make an individual’s attitudinal inconsistency salient [35]. Interestingly, the predictors did not significantly impact on felt ambivalence at high potential ambivalence. This could indicate a primacy of potential ambivalence in leading to felt ambivalence regardless of the aforementioned individual differences. Thus, another important explanation could be that the individual differences influence the palliation of felt ambivalence only at low levels of potential ambivalence. For example, a male gender might facilitate averting experiences of felt ambivalence at some levels of potential ambivalence but not at high potential ambivalence. At high levels of potential ambivalence, it might become too difficult to avert felt ambivalence. Interestingly, sociodemographic variables did not significantly moderate the effects of meat ambivalence on behavioral change in the present set of studies. Gender, for example, therefore seems to influence the extent to which people experience conflict rather than how they react to it. This could arguably explain why previous research has not consistently found (the absence of) gender differences in the use of coping mechanisms related to meat consumption (e.g., [118,119]).

Based on the present research, a novel intervention paradigm for empowering decision competence on healthy and sustainable food choices could encourage individuals to reflect on their chronic ambivalence about meat. The experimental manipulation of felt ambivalence in Study 2 and 3 elicited behavioral change merely by making felt ambivalence salient. This finding suggests that introspective ambivalence elicitation can complement persuasive intervention approaches that are commonly employed to facilitate meat reduction [120,121,122]. Specifically, the efficacy of interventional messages could potentially be enhanced by prompting individuals to reflect on their personal conflicts between preexisting incongruent evaluations of meat consumption. This prompt may motivate them to reconcile the incongruent beliefs and obtain a non-ambivalent attitude, such as through acquiring information on the practicalities of plant-based diets (e.g., [10]). Yet another downstream benefit of ambivalence-induced effortful elaboration is that it fosters more persistent attitudinal and behavioral change [68,114]. As our data showed participants to hold opposing evaluations about health benefits and risks related to meat (see Supporting Information for the Ancillary Study), one major application concerns health messages that can include an elicitation of personally held ambivalence in addition to information that helps in resolving conflict about health implications of meat consumption.

Yet, at first glance, the finding that felt ambivalence can motivate meat reduction seems at odds with research on the “meat paradox” [47], which has drawn on cognitive dissonance theories [123] to explain how meat consumption is maintained in light of detrimental consequences [46,124]. Research on ambivalence and dissonance reflect methodologically and conceptually distinct frameworks (see [125]). For one, research on meat-related dissonance focuses on situational conflicts between behavior and cognition, such as by presenting participants with messages about critical issues related to meat while increasing the salience of meat commitment (e.g., [106]). In our set of studies, in contrast, felt ambivalence reflects a chronic awareness of experiences of conflict between stably held but incongruent evaluations of meat. While we expect that most but not all people who are chronically ambivalent will reduce their meat consumption, the present research adds to previous cross-sectional studies (e.g., [18,57]) by investigating the causal direction and mechanisms involved in why ambivalence is associated with, on average, less meat consumption. In sum, our proposed model is a step toward understanding the positive effects of one group of meat-related conflicts in motivating people to eat less meat. Future research could explore the conditions under which the consequences of ambivalence and dissonance on meat consumption overlap or diverge.

A limitation of Study 2 and 3 is that we utilized intended meat reduction as an outcome but not actual meat consumption. However, experimentally induced behavioral intentions are an important predictor of behavior (see [96] for a meta-analysis), and Study 3 supported the pattern of findings employing a performance-based outcome measure of charitable donation behavior and an information selection task, whereas Study 1 employed a robust six-day diary of meat consumption.

A promising avenue for future research on ambivalence-motivated meat reduction concerns potential roles of cultural differences. On the one hand, the prevalence of ambivalence toward meat consumption could depend on cultural differences, such as in relation to the impact of masculinity and food proscriptions on attitudes toward meat [36,39]. Moreover, the attitudinal drivers of meat ambivalence could partially differ across cultures. For example, the stigmatization of meat consumption in India [36] might increase the centrality of sociability as a driver of meat ambivalence, whereas frequent exposure to animal slaughter in Ecuador [38] could attenuate the centrality of associations to animals in meat ambivalence. A comprehensive approach to gain insight into the drivers of meat ambivalence and the role of cultural differences is attitude network analysis [125,126], which can estimate the interrelations between large sets of variables and test for differences in the interrelations across samples (e.g., [127,128]). A network analysis could also investigate the extent to which the drivers of meat ambivalence are differentially related to ambivalence-motivated meat reduction. Specifically, the centrality of the drivers could arguably predict how people react to meat ambivalence, and the centrality of the drivers should depend on country differences. For instance, the extent to which people cope with sociability-related meat ambivalence through meat reduction could depend on cultural differences in social norms. Differences in social norms might, however, be less likely to influence coping with animal-related ambivalence in comparison to sociability-related ambivalence.

Future research could also investigate ambivalence-motivated meat reduction over time. There are cross-national differences both in meat ambivalence [3] and the directions of meat consumption trends [1,129]. Moreover, in Switzerland, for example, people are increasingly concerned about the effects of meat consumption [5], whereas the stigmatization of meat consumption has arguably been decreasing in India [36,130]. These cross-national and temporal differences yield a promising opportunity to explore also further questions, including potentially differential roles of the drivers of ambivalence in ambivalence-motivated meat reduction.

While the experience of evaluative inconsistency arguably tends to be universally aversive [25,123,125,131], the motivational consequences of this ambivalent discomfort on meat reduction could arguably be attenuated by cultural differences. Cultural differences such as in tolerance for inconsistency [132] or traditional eating [133] could influence the extent to which ambivalence toward meat consumption is experienced as aversive, with downstream consequences on the extent to which ambivalence motivates people to eat less meat. For instance, one could argue that ambivalence-motivated meat reduction is more pronounced in people who score low in tolerance for inconsistency, like people from Western cultures compared to people from East Asian cultures. As such, future research needs to explore ambivalence-motivated meat reduction in a broader array of cultural contexts.

The present research sheds light on the association of ambivalence with behavioral change in the context of meat consumption, revealing how the need to cope with meat-related ambivalence can motivate meat reduction. Our findings add to the growing body of literature on why some people eat less meat by highlighting the importance of the motivational consequences of ambivalence.

## Figures and Tables

**Table 1:** Characteristics of the initial and final samples in Study 1.

Variable		Final Sample	Initial Sample
*M*_age_ (*SD*_age_)		52.49 (13.62)	51.09 (15.14)
Education ^†^		49%	48%
Female		48%	50%
Region	North	5%	6%
	East	13%	13%
	West	51%	51%
	South	30%	32%

Note. ^†^ Education (dummy-coded) refers to the percentage of people with at least a qualification for university entrance.

**Table 2:** Predictive correlates of felt ambivalence about meat consumption as indicated by Pearson correlation tests, except for point-biserial correlation tests with gender and part-time employment.

Variable	*r*	*p*
Gender	0.16	<0.001
Social context	0.23	<0.001
Student	0.14	0.001
Age	−0.13	0.003
Part-time employment	0.10	0.013
Potential ambivalence	0.41	<0.001

Note. *N* = 555. For gender (1—*female*, 0—*male*) and age, *N* = 553. Part-time employment was coded as 1—*part-time employment* and 0—*other*.

**Table 3:** Multiple regression results for predicting meat consumption frequency from felt ambivalence (model 1) and from felt ambivalence and behavioral control (model 2).

Variable	Model 1			Model 2		
	*B*	*SE*	β	*B*	*SE*	β
Constant	0.59 **	0.02		0.62 **	0.03	
Felt ambivalence	−0.04 **	0.01	−0.18 **	−0.03 **	0.01	−0.16 **
Behavioral control				−0.03 *	0.01	−0.10 *
*R* ^2^	0.03 **			0.04 **		
Δ*R*^2^	0.03 **			0.01 *		

* *p* < 0.05. ** *p* < 0.001.

## Data Availability

Data files, supporting information, and preregistrations are openly available in OSF at osf.io/7w69j (last accessed on 13 January 2022), save the dataset from Study 1, which will be uploaded once an unrelated part of the dataset has been published in a separate article.

## Supplementary Materials

Supporting information can be downloaded at https://osf.io/a74cv/ (last accessed on 13 January 2022).
